# A novel gingipain regulatory gene in *Porphyromonas gingivalis* mediates host cell detachment and inhibition of wound closure

**DOI:** 10.1002/mbo3.1128

**Published:** 2020-10-13

**Authors:** Hongyan Liu, Lijia Huang, Yanling Cai, Floris J. Bikker, Xi Wei, Dong Mei Deng

**Affiliations:** ^1^ Department of Operative Dentistry and Endodontics Hospital of Stomatology Sun Yat‐sen University Guangzhou China; ^2^ Guangdong Province Key Laboratory of Stomatology Guangzhou China; ^3^ Department of Oral Biochemistry Academic Centre for Dentistry Amsterdam Free University and University of Amsterdam Amsterdam The Netherlands

**Keywords:** biofilm, epithelial cell detachment, gingipain, *pgn_0361*, *Porphyromonas gingivalis*, wound closure

## Abstract

The black pigmentation‐related genes in *Porphyromonas gingivalis* are primarily involved in regulating gingipain functions. In this study, we identified a pigmentation‐related gene, designated as *pgn_0361*. To characterize the role of *pgn_0361* in regulating *P. gingivalis*‐mediated epithelial cell detachment and inhibition of wound closure, PgΔ0361, an isogenic *pgn_0361*‐defective mutant strain, and PgΔ0361C, a complementation strain, were constructed using *P. gingivalis* ATCC 33277. The gingipain and hemagglutination activities, as well as biofilm formation, were examined in all three strains. The effect of *P. gingivalis* strains on epithelial cell detachment was investigated using the HO‐1‐N‐1 and Ca9‐22 epithelial cell lines. The inhibition of wound closure by heat‐killed *P. gingivalis* cells and culture supernatant was analyzed using an *in vitro* wound closure assay. Compared to the wild‐type strain, the PgΔ0361 strain did not exhibit gingipain or hemagglutination activity but exhibited enhanced biofilm formation. Additionally, the PgΔ0361 strain exhibited attenuated ability to detach the epithelial cells and to inhibit wound closure *in vitro*. Contrastingly, the culture supernatant of PgΔ0361 exhibited high gingipain activity and strong inhibition of wound closure. The characteristics of PgΔ0361C and wild‐type strains were comparable. In conclusion, the *pgn_0361* gene is involved in regulating gingipains. The PGN_0361‐defective strain exhibited reduced virulence in terms of epithelial cell detachment and inhibition of wound closure. The culture supernatant of the mutant strain highly inhibited wound closure, which may be due to high gingipain activity.

## Graphical abstract

We identified a pigmentation‐related gene *pgn_0361*. The *pgn_0361*‐defective strain of *Porphyromonas gingivalis* showed a reduced ability of wound closure inhibition and detachment of oral epithelial cells. The culture supernatant of *pgn_0361*‐defective strain showed higher gingipain activity and enhanced wound closure inhibition. The introduction of *pgn_0361* into the defective strain reversed that trait.

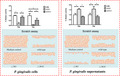

## 1 INTRODUCTION

The colonies of several oral bacterial species, including *Porphyromonas gingivalis* and *Prevotella* spp., exhibit black pigmentation on the blood agar medium. The black pigmentation phenotype is associated with the initiation and development of various infections, including periodontitis and endodontic infections (Dahlen, 1993; Matto et al., 1997). In a mouse infection model, the administration of black‐pigmented *P. gingivalis* was lethal, whereas the administration of a spontaneously pigment‐less *P. gingivalis* variant did not cause lethality (McKee et al., 1988). This indicated that the pigment‐less phenotype of *P. gingivalis* is associated with reduced virulence.

Various studies on *P. gingivalis* have revealed that the black colony pigmentation is caused due to heme accumulation on the cell surface. *P. gingivalis* acquires iron through gingipain‐mediated hemoglobin digestion in the microenvironment. The hemagglutinins (such as HagB and HagC) facilitate the acquisition of heme through erythrocyte binding, allow the gingipains to release the heme moiety from the hemoglobin molecule, and uptake by the bacterial (Connolly et al., 2017). Meanwhile, the heme‐binding domains (HA2) of gingipain and the adhesion domains of hemagglutinins are both able to bind heme and hemoglobin, facilitating the heme deposition (Smalley & Olczak, 2017). Heme and peptides that are released after digestion are used by the cells as nutrients or stored on the cell surface, which leads to a black pigmentation phenotype (Shoji et al., 2002; Smalley et al., 1998). Based on their specificity, gingipains can be classified as arginine‐specific proteases (RgpA and RgpB) and lysine‐specific proteinases (Kgp) (Smalley & Olczak, 2017). Interestingly, all known pigmentation‐related genes are functionally linked to gingipains, especially *kgp*, or genes involved in the regulation, transportation, and attachment of gingipains (Sato et al., 2009).

*Porphyromonas gingivalis* is reported to have a complex gingipain regulation system, which involves several genes or gene clusters (Nakayama et al., 2015). Several studies have focused on the genes and gene clusters related to gingipain function (Abaibou et al., 2001; Burgess et al., 2002; Vanterpool et al., 2004). However, there are limited studies that have evaluated the involvement of gingipain in the interaction between *P. gingivalis* and host cells. Gingipains are reported to be key mediators in *P. gingivalis*‐induced inflammation and tissue damage. Previous studies have demonstrated that gingipains can degrade various human cellular proteins, including cytokines, integrins, and collagen, or can alter cellular signal transduction (Nakayama & Ohara, 2017). Gingipains are also involved in detaching the epithelial cells from the connective tissues of the gingiva (Baba et al., 2001) and inhibiting the wound healing process (Laheij et al., 2015). However, it is not clear whether the gingipain‐regulating genes affect the interaction between *P. gingivalis* and host solely through regulation of gingipain function or in combination with other functions of the individual gene.

The screening of the non‐pigmented *P. gingivalis* strains in a transposon mutagenesis library revealed several pigmentation‐related genes, such as *kgp* and *gtfB* (Chen et al., 2000; Yamaguchi et al., 2010). However, the two transposon mutagenesis systems that were screened (Tn*4351* and Tn*4400*) have several disadvantages. In both systems, the elements are inserted preferentially into the “hot‐spots” in the genome, which limits the distribution of interrupted genes. Additionally, introducing Tn*4351* into *P. gingivalis* may result in duplication and transposition of the endogenous insertion element (Simpson et al., 1999). Therefore, the complexity of *P. gingivalis* genomic rearrangement after Tn*4351* transposition restricts its use in transposition mutagenesis. The Himar 1 Mariner mini‐transposon system was developed in 2012, which addressed the limitations of previous systems. This system allows a single, stable transposition event. This system has been successfully applied to identify the genes that are essential for the growth of *P. gingivalis* (Klein et al., 2012) and to identify pigmentation‐related genes (Klein et al., 2017).

In this study, we generated a transposon library of the *P. gingivalis* ATCC 33277 strain using the Mariner system independently to isolate several non‐pigmented mutants. Interestingly, we also identified an insertion within the *pgn_0361* gene, which was previously characterized as the pigmentation‐related gene by Klein et al. (Klein et al., 2017) using the same Mariner system. This study aimed to investigate the involvement of *pgn_0361* in the interaction between *P. gingivalis* and host cells. Specifically, the role of *pgn_0361* in regulating the *P. gingivalis*‐mediated epithelial cell detachment and inhibition of wound healing was examined.

## 2 MATERIALS AND METHODS

### 2.1 Bacterial strains and growth conditions

*Porphyromonas gingivalis* ATCC 33277 was used as the parental strain and all mutants were constructed based on this genetic background. The *P. gingivalis* strains were routinely cultured on 5% sheep blood tryptic soy agar plates (Becton Dickinson B.V., Breda, the Netherlands) supplemented with hemin (5 μg/mL) and menadione (1 μg/mL) (BA plates) under anaerobic conditions (80% N_2_, 10% H_2_, and 10% CO_2_) at 37°C. The mutant strains (transposon mutants) were selected in the presence of 50 μg/mL gentamicin and 5 µg/mL erythromycin. The complement mutants were selected in the presence of 50 μg/mL gentamicin and 1 µg/mL tetracycline.

*Escherichia coli* S17‐1 was used for maintenance, construction of plasmids, and conjugation. The pSAM_Bt plasmid containing the *bla*, *ermG*, and *himar1c9a* genes was obtained from Dr. Brian Klein. S17‐1 was cultured in Luria–Bertani (LB) broth or LB agar. Ampicillin (100 µg/ml) was added to the medium when necessary.

### 2.2 Transposon mutagenesis

The Mariner‐based transposon mutants were constructed by conjugation, following the protocol described by Klein et al. (Klein et al., 2012). Briefly, the *P. gingivalis* cells (OD_600_ = 0.5–1) were mixed with the culture of *E. coli* S17‐1 (OD_600_=0.5) harboring the pSAM_Bt plasmid. The mixture was plated on the BA plates containing 50 μg/ml gentamicin and 5 µg/ml erythromycin. The erythromycin‐resistant transposon mutants were selected.

### 2.3 Construction of the *P. gingivalis* mutant strain

To generate the mutant strain, the *pgn_0361* coding region was replaced with the *ermF* gene using the PCR ligation mutagenesis method as described previously (Lau et al., 2002). The genomic DNA (gDNA) of the *P. gingivalis* ATCC 33277 strain was isolated using the GeneJet genomic DNA purification kit (ThermoFisher, Waltham, MA, USA) and was used as a template for PCR. The flanking regions of the PGN_0361 gene were amplified from gDNA using the following two PCR primer pairs: 361uf/361ur (361uf 5′‐TCTGCAAGGCTACGACTATCT‐3′; 361ur 5′‐ACTAGTGAGAAGGCACTGTTCCAAGA‐3′) and 361df/361dr (361df 5′‐GGCGCGCCTGATACATCTTCCCTTAGAAAG‐3′; 361dr 5′‐GCAGCCATACACCTGCTCATT‐3′). The erythromycin resistance gene (Em gene) was amplified from the pEP4351 plasmid using the following primer pair: Emf/Emr (Emf 5′‐ACTAGTGTTTCCGCTCCATCGCCAATTTGC‐3′; Emr 5′‐GGCGCGCCCGATAGCTTCCGCTATTGC‐3′). These three PCR fragments were digested with restriction enzymes SpeI and AscI (indicated by the underlined sequences) and ligated for 16 h at 4 °C. The ligated fragment was used as the template for the amplification of the mutant construct using the 361uf/361dr primer pair. The resulting PCR product was purified and transformed directly into the *P. gingivalis* cells by electroporation. The mutant strain was obtained via a double crossover. The insertion of the Em gene was verified by PCR and Sanger sequencing. The resulting strain was designated as PgΔ0361.

### 2.4 Complementation of the PgΔ0361 mutant strain

To complement the function of *pgn_0361*, a region containing the complete PGN_0361 sequence (including a 105 bp upstream region) was amplified from the gDNA of the ATCC 33277 strain by PCR. The complete fragment was cloned into the shuttle vector, pT‐COW. The primer sequences used were as follows: 361F_com, 5′‐GCATGCCGGTAGGAGAGGACGACCTCTTGT‐3′; 361R_com, 5′‐GTCGACCATGGCGATCCGAATACGATCA‐3′. The resulting plasmid was transformed into *E. coli* S17‐1 and the recombinant colonies were selected based on ampicillin resistance. The plasmid was introduced into the corresponding PgΔ0361 strain by conjugation. The presence of the newly constructed plasmid was confirmed by PCR. The PgΔ0361 strain containing the pT‐COW‐derived plasmid was designated as PgΔ0361C.

### 2.5 Characterization of the mutant PgΔ0361 strain

The mutant PgΔ0361 strain was characterized along with the corresponding wild‐type ATCC 33277 strain and PgΔ0361C strain. The hemagglutination and gingipain activities, as well as the ability to form biofilms, were investigated in all three strains.

### 2.6 Gingipain activity

The late exponential culture (2 ml; OD_600_ = 1) of *P. gingivalis* strains was subjected to centrifugation at 21,200 *g* for 2 min. The supernatant was collected, and the pellet was resuspended in Tris‐buffered saline (TBS, 50 mM Tris, 150 mM NaCl, pH 7.6). The supernatant was filter‐sterilized using a 0.2‐μm filter before further analysis. The gingipain activities of the cell pellet and supernatant were quantified using a fluorescence resonance energy transfer (FRET) assay (Kaman et al., 2012). The specific fluorogenic probes used for detecting the activities of Rgp and Kgp were BikKam15 ([FITC]‐Phe‐Arg‐[KDbc]) and BikKam14 ([FITC]‐Lys‐Lys‐[KDbc]), respectively. The final concentrations of BikKam15 and BikKam14 were 32 μM and 16 μM, respectively. The samples were incubated with the probes in TBS at 37°C for 1 h. The fluorescence intensity (FI) of the mixture was recorded at an excitation wavelength of 485 nm and an emission wavelength of 530 nm in a fluorescence microplate reader (SpectraMax^®^, Molecular Devices, San Jose, CA, USA). The relative gingipain activity was calculated by dividing the FI value of each sample by the average FI value of the wild‐type group. Three experiments were performed with duplicate samples for each group.

### 2.7 Hemagglutination activity

The hemagglutination activities of all three strains were examined following previously described protocols (Muthiah et al., 2013; Yamaguchi et al., 2010). Briefly, the OD_600_ of the late exponential *P. gingivalis* cells was adjusted to 1 in phosphate‐buffered saline (PBS) and serially diluted in a round‐bottom 96‐well plate (100 μl/well). The *P. gingivalis* cells were incubated with an equal volume of 1% PBS‐washed sheep erythrocytes (Biotrading, Mijdrecht, the Netherlands) at 22°C for 3 h. Hemagglutination was visually assessed. The last dilution that exhibited complete hemagglutination was considered as the titer.

### 2.8 Biofilm formation

Biofilms were grown in a 96‐well active attachment model (Li et al., 2014). The 24‐h pre‐culture of each *P. gingivalis* strain was diluted (1:20) in fresh brain–heart infusion (BHI) broth supplemented with hemin (5 μg/ml) and menadione (1 μg/ml) (BHIHM) and pipetted into a 96‐well plate (200 μl/well). The lid with 96 pegs (NuncTM, Roskilde, Denmark) was placed into the microtiter plate to allow biofilm formation. The pegs were transferred to fresh BHIHM after 24 h. The biomass of 48‐h *P. gingivalis* biofilms was quantified by crystal violet assay (Li et al., 2014). Three experiments were performed with quadruplicate samples from each group per experiment.

### 2.9 Eukaryotic cell culture

The human oral buccal epithelial cell line (HO‐1‐N‐1) (Laheij et al., 2015) and gingival epithelial cell line (Ca 9‐22) (Japanese Collection of Research Bioresources) were cultured in tissue culture flasks containing Dulbecco's modified Eagle's medium/Nutrient Mixture F‐12 (DMEM/F‐12; Life Technologies, Waltham, MA, USA) supplemented with 10% fetal calf serum (Hyclone, Logan, UT, USA) and 1% antibiotics (penicillin, streptomycin, and fungizone, Sigma, St. Louis, MO, USA) in a humidified atmosphere at 37°C and 5% CO_2_.

### 2.10 Detachment assay

The ability of the three *P. gingivalis* strains to detach the HO‐1‐N‐1 and Ca9‐22 cells was evaluated using a detachment assay (Huang et al., 2016). The confluent (80%) cell cultures were trypsinized and resuspended in the DMEM/F‐12 medium supplemented with 10% fetal calf serum. The resuspended cells were seeded in a 96‐well plate at a cell density of 3.5 × 10^4^ cells/well for 24 h. The cells were washed with PBS twice, followed by the addition of 100 μl *P. gingivalis* cultures at a multiplicity of infection (MOI, the ratio of bacteria: cells) of 50,000 or only 100 μl medium (control). The *P. gingivalis* cultures were prepared by centrifuging the late exponential cultures at 5,000 *g* for 15 min. The cell pellet was resuspended with the culture medium. The co‐culture was incubated at 37°C and 5% CO_2_ for 4 h. Next, all cells were washed twice with 200 μl PBS, followed by fixation with 4% formaldehyde at 22°C for 2 h. The cells were stained with Alexa Fluor 488 phalloidin (Life Technologies, Waltham, MA, USA), following the manufacturer's protocol. Four images of each well were captured under a 5X objective lens using an inverted digital phase‐contrast microscope EVOS FL (Advanced Microscopy Group, USA). The relative cell coverage of each well was calculated by averaging the percentage cell coverage of four images. The level of detachment was determined based on the cell coverage area in the test group relative to that in the control group (without the addition of bacteria), which was calculated by dividing the coverage area in the test group by the average coverage area percentage under control conditions.

### 2.11 In vitro wound closure assay

The effects of the three *P. gingivalis* strains on the wound healing process were evaluated using an *in vitro* wound closure assay. Both heat‐inactivated *P. gingivalis* cells and supernatants collected from the late exponential *P. gingivalis* cultures (OD_600_ = 1) were used to challenge the host cells. The supernatants were filter‐sterilized before use.

The heat‐inactivated *P. gingivalis* cells were prepared according to the protocol described by Laheij et al. (2015). The late exponential *P. gingivalis* cells were washed twice in PBS (pH 7.4) and resuspended in keratinocyte serum‐free medium (SFM; ThermoFisher, Waltham, MA, USA). The resuspended cells were incubated at 60°C in a water bath for 60 min. The killing of bacterial cells was confirmed by the absence of *P. gingivalis* growth on the BA plates.

The wound closure assays were performed as described previously (Laheij et al., 2015). The HO‐1‐N‐1 cells were seeded in a 24‐well plate at a concentration of 3 × 10^5^ cells/well for 6 h. An artificial wound was created by scraping the confluent cell monolayer using a sterile 1‐ml micropipette tip. The epithelial cells were treated with *P. gingivalis* culture supernatant or heat‐inactivated *P. gingivalis* cells. The supernatant was diluted three times with SFM before addition to the wells. The *P. gingivalis* growth medium (BHIHM) was diluted similarly to serve as a negative control for the supernatant groups. The heat‐inactivated *P. gingivalis* cells at MOIs of 10, 100, and 1,000 were tested. The SFM was used as a negative control for the heat‐inactivated groups. The co‐cultures were incubated in a humidified atmosphere at 37°C and 5% CO_2_ in a Bioflux system (Fluxion).

The cells in the middle of the well were simultaneously imaged at a magnification of 50X after the addition of *P. gingivalis* challenges. The images were captured at 2‐h intervals for 20 h. The area of the scratch in each image was calculated using the ImageJ software (version 1.49, U.S. National Institutes of Health).

The closure percentage of the scratch was calculated as follows: 100 − [(surface area of the scratch at 20 h/surface area of the scratch at 0 h) × 100]. To minimize the differences in wound closure between experiments, the closure of the scratch relative to control (relative closure) was calculated as follows: the percentage of the closure of the scratch/the average percentage of the closure of the scratch under control conditions (SFM) × 100%. The experiment was repeated four times with 2–3 replicates per condition per experiment.

### 2.12 Statistical analysis

The data were analyzed using the Statistical Package for Social Science (SPSS, version 23.0). One‐way analysis of variance (ANOVA), followed by Bonferroni post hoc test was used to compare the gingipain activity and host cell detachment data of the wild‐type, PgΔ0361, and PgΔ0361C *P. gingivalis* strains. The Kruskal–Wallis test, followed by Mann–Whitney U post hoc test, was used to compare the biomass of three *P. gingivalis* strains as not all data met the prerequisites of normal distribution and equality of variance. The α value was set at 0.0167 (0.05/3). To evaluate the influence of the PGN_0361 deletion on wound healing ability, the relative cell coverage area was analyzed using a two‐way ANOVA, followed by the Bonferroni test. The difference was considered statistically significant when the *p*‐value was less than 0.05.

## 3 RESULTS

### 3.1 Identification of genes associated with non‐pigmentation

In total, 400 transposon mutants were screened on the BA agar plates for colony pigmentation. Among these mutants, 11 colonies displayed altered pigmentation. To locate the transposon insertion in the genome, the gDNA of these 11 mutants with altered pigmentation was extracted and used as a template in a nested semi‐random PCR (Klein et al., 2012). The PCR products were subjected to sequencing analysis, which revealed one insertion site in the *pgn_0361* gene that encodes a putative glycosyltransferase (family 2). Another mutant contained an insertion site in the *pgn_1240* gene which encodes a putative glycosyltransferase (group 1). The rest mutants contained insertions sites in either the *rgpA*/*B* gene or *kgp* gene.

### 3.2 Characterization of the mutant PgΔ0361 strain

**FIGURE 1:**
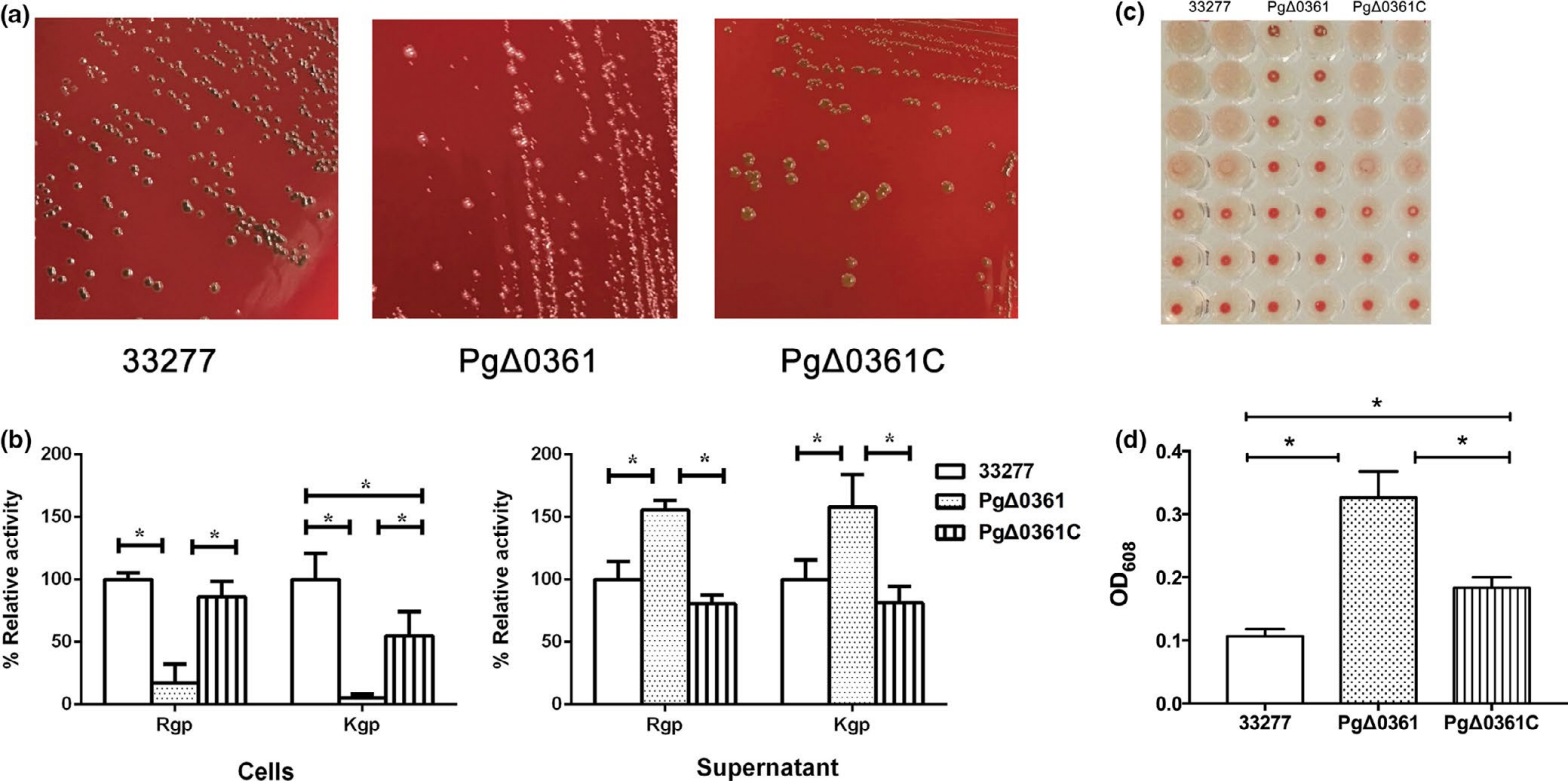
Characterization of the mutant PgΔ0361 strain. (A) The colonies of *Porphyromonas gingivalis* strains. The *P. gingivalis* wild‐type ATCC 33277 strain is indicated as 33277; (B) the percentage Rgp and Kgp activities of the supernatant and cell pellet of *P. gingivalis* strains. The activity levels were normalized to the activities exhibited by the supernatant and cell pellet of the ATCC 33277 strain (100%). *p < 0.05. (C) Hemagglutination activities of *P. gingivalis* strains. The blank contained only the medium. (D) Biofilm formation by ATCC 33277, PgΔ0361, and PgΔ0361C strains. *p < 0.0167.

We constructed a PgΔ0361 strain by gene‐directed mutagenesis and a PgΔ0361C strain by introducing the wild‐type *pgn_0361* gene into the PgΔ0361 strain. The PgΔ0361, PgΔ0361C, and wild‐type strains were characterized (Figure 1). The colonies of PgΔ0361 did not exhibit black pigmentation on the BA plate, whereas those of PgΔ0361C exhibited the same black pigmentation as the wild‐type strain (Figure 1A). The Rgp and Kgp activities of the cell pellets and culture supernatants of the three *P. gingivalis* strains are presented in Figure 1B. The Rgp and Kgp activities of the PgΔ0361 cell pellets were 17 ± 15% and 5 ± 3%, respectively, which were significantly lower than those of the wild‐type and PgΔ0361C strains (*p* < 0.05). The Rgp and Kgp activities of the PgΔ0361 cell culture supernatant were 155 ± 7% and 158 ± 26%, respectively, which were significantly higher than those of the wild‐type and PgΔ0361C strains (*p* < 0.05). The hemagglutination activity and biofilm formation of the strains are shown in Figure 1C and 1D, respectively. The PgΔ0361 strain did not exhibit hemagglutination activity. The wild‐type and PgΔ0361C strains exhibited similar hemagglutination activities. The biomasses of the PgΔ0361 biofilms were 2‐fold higher than those of the wild‐type biofilms. The biofilm biomass of the PgΔ0361C strain was lower than that of the PgΔ0361 strain and was significantly higher than that of the wild‐type strain (*p* < 0.0167).

### 3.3 Role of *pgn_0361* in epithelial cell detachment and wound closure

**FIGURE 2:**
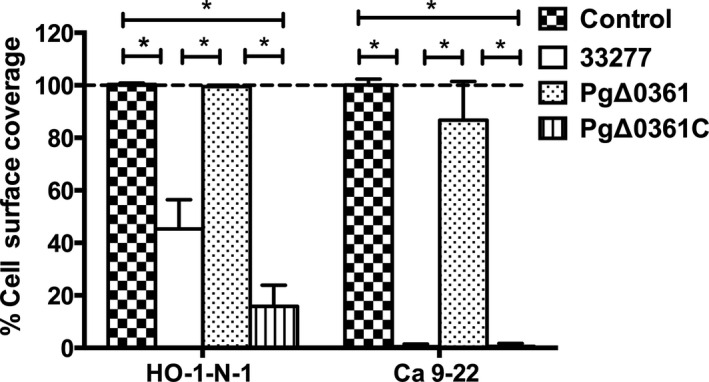
The percentage of HO‐1‐N‐1 and Ca9‐22 cell surface area coverage after co‐incubation with various *Porphyromonas gingivalis* strain cultures for 4 h. The *P. gingivalis* cultures with the multiplicity of infection (MOI) of 50,000 were used. The cells treated with the medium were used as a control. *p < 0.05.

**FIGURE A1:**
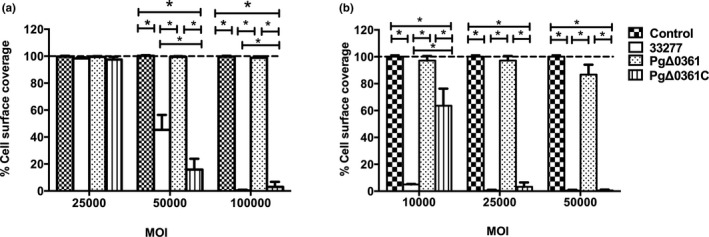
The percentage of HO‐1‐N‐1 (A) and Ca9‐22 (B) cell surface area coverage after co‐incubation with various *Porphyromonas gingivalis* strain cultures for 4 h. The *P. gingivalis* cultures of various MOIs were used. The cells treated with the medium were used as a control. *p < 0.05.

Figure 2 shows the ability of *P. gingivalis* cell resuspensions to detach the epithelial HO‐1‐N‐1 and Ca9‐22 cells. At MOI 50,000, the wild‐type strain detached 50%–100% of the epithelial cells from the well bottom, whereas the PgΔ0361 strain did not cause any visible detachment. The PgΔ0361C strain could detach the epithelial cells. Moreover, the strength of attachment was different between the two epithelial cell lines tested. The wild‐type strain at MOI 50,000 caused 100% detachment of Ca 9–22 cells and only 50% detachment of HO‐1‐N‐1 cells. We also evaluated the detachment abilities of three *P. gingivalis* strains using various MOIs. The detachment of Ca 9–22 cells was further examined with MOIs of 10,000 and 25,000, whereas the detachment of HO‐1‐N1 cells was further examined with MOIs of 25,000 and 100,000. Figure A1 presents the cell detachment results obtained from all MOIs. Generally, increasing cell detachment could be observed as the MOIs of *P. gingivalis* wild‐type and complement strains increased. However, the mutant strain was unable to detach any tested epithelial cells irrespective of MOI values.

**FIGURE 3:**
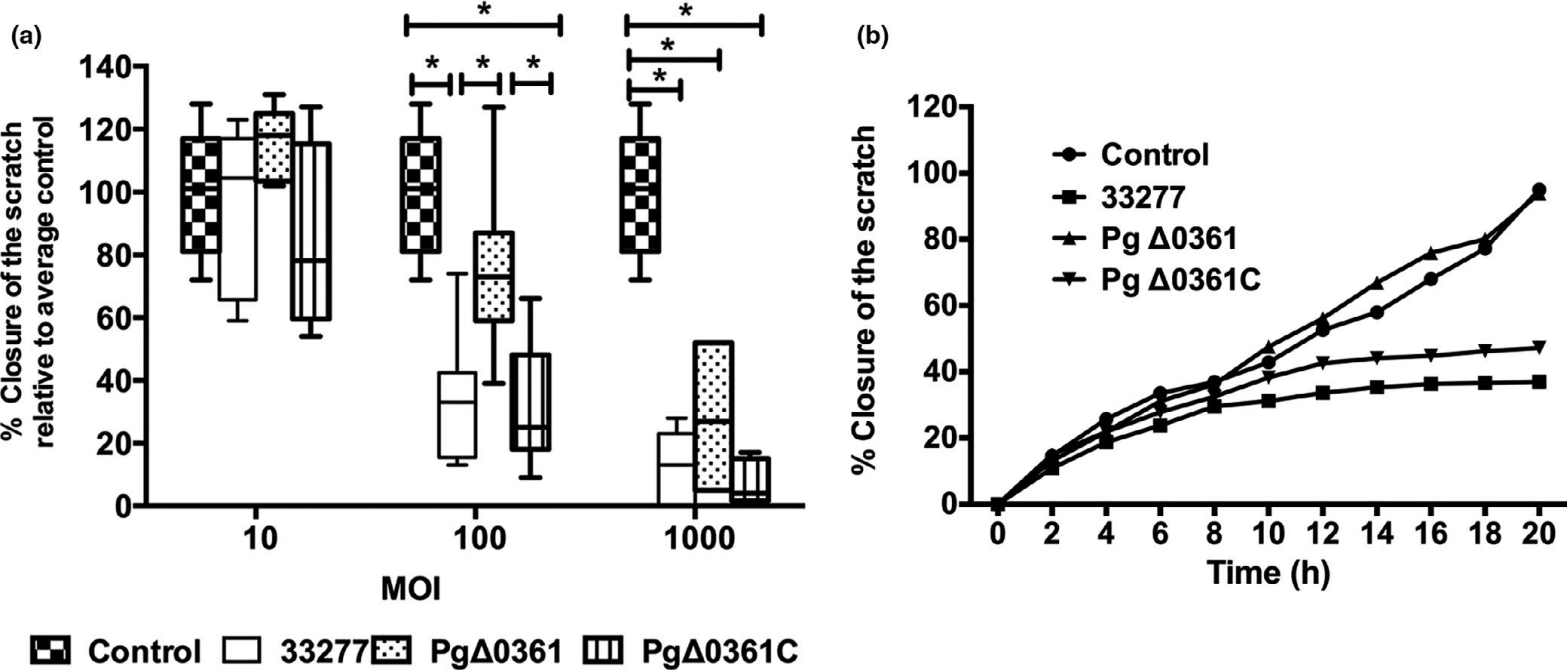
Wound closure in the oral buccal epithelial cells challenged with heat‐inactivated *Porphyromonas gingivalis* strains. (A) Mean relative closure (±*SD*) from all biological replicates of scratches in the oral buccal epithelial cells challenged with heat‐inactivated *P. gingivalis* strains after 20 h. (B) Time curve of percentage closure of a scratch in the oral buccal epithelial cells challenged with heat‐inactivated *P. gingivalis* strains at MOI 100. The cells treated with the medium were used as a control. *p < 0.05.

In the wound closure assay, only the HO‐1‐N‐1 cells were tested as they exhibit strong adhesion to the surfaces of the polypropylene 24‐well plate. As shown in Figure 3A, the heat‐killed *P. gingivalis* strains dose‐dependently inhibited the epithelial cell migration after co‐incubation for 20 h. However, the levels of epithelial cell migration inhibition varied among the tested strains. For example, the PgΔ0361 strain exhibited significant inhibition of wound closure at the highest MOI tested (MOI 1,000), whereas the other two strains exhibited strong inhibition at MOI of 100. Additionally, the PgΔ0361 cells at the highest MOI exhibited non‐significantly lesser inhibition of wound closure than the wild‐type and PgΔ0361 strains (*p* > 0.05).

**FIGURE 4:**
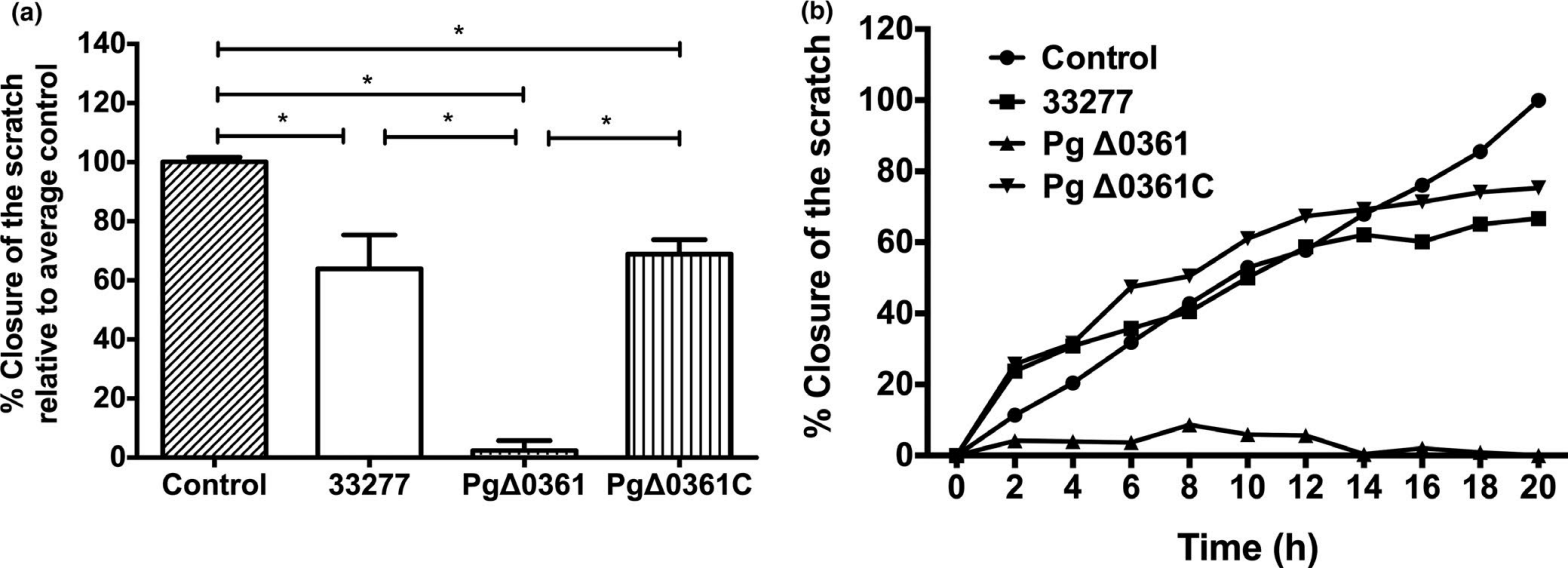
Wound closure in the oral buccal epithelial cells challenged with supernatant of *Porphyromonas gingivalis* strains. (A) Mean relative closure (±*SD*) from all biological replicates of scratches in the oral buccal epithelial cells challenged with supernatants of the *P. gingivalis* strains after 20 h. (B) Time curve of percentage closure of a scratch in HO‐1‐N‐1 cells challenged with culture supernatants of *P. gingivalis* strains. *P. gingivalis* growth medium BHIHM was diluted three times with SFM to serve as a negative control for the supernatant groups. *p < 0.05.

The culture supernatant of PgΔ0361 exhibited almost complete inhibition of wound closure after 20 h (Figure 4A). The supernatants of the other two strains exhibited limited inhibition of wound closure. Additionally, treatment with the supernatant negative control, which was prepared by diluting the BHIHM 3 times with the SFM medium, delayed the wound closure. After 20 h, the wound closure of this group was only 50% of that in the SFM control group (data not shown).

The dynamic effects of *P. gingivalis* heat‐killed cells (MOI 100) and supernatants on wound closure are shown in Figures 3B and 4B, respectively. The percentage of wound closure was plotted against time at 2‐h intervals. In the medium control group, the scratch closure rate increased with time following a linear pattern. The heat‐killed wild‐type and PgΔ0361C strains (Figure 3B) exhibited marked inhibition of wound closure after 10–12 h of co‐incubation. However, the culture supernatant of the heat‐killed wild‐type and PgΔ0361C strains exhibited clear inhibition of wound closure at 16–18 h (Figure 4B). The culture supernatant of PgΔ0361 completely inhibited the wound closure during the 20‐h co‐incubation period (Figure 4B).

## 4 DISCUSSION

In this study, we identified the black pigmentation‐related gene (*pgn_0361*) in a transposon mutant library of *P. gingivalis* ATCC 33277, which was generated using the Mariner transposon system. The *pgn_0361* insertion mutant was isolated by other researchers using the same method (Klein et al., 2017; Naito et al., 2019). Klein et al. reported that this gene is involved in pigmentation (Klein et al., 2017). The *pgn_0361* gene deletion strain generated in this study exhibited altered phenotypes, such as non‐pigmented colonies on the BA plate and impaired gingipain activities. Additionally, the mutant strain exhibited decreased hemagglutinin activity, which was consistent with the results of a previous study (Klein et al., 2017).

So far, fifteen genes have been reported to be associated with black pigmentation in *P. gingivalis* (Abaibou et al., 2001; Hasegawa et al., 2003; Muthiah et al., 2013; Okamoto et al., 1998; Saiki & Konishi, 2007; Sato et al., 2009; Shoji et al., 2002; Yamaguchi et al., 2010). The deletion of most of these genes, except *ugdA*, results in impaired hemolytic and hemagglutinin activities (Sato et al., 2009). The reduction in hemagglutinin activity of the *pgn_0361* mutant strain indicated that the *pgn_0361* gene shared general functions of the pigmentation‐related genes. Of the 15 genes, two are putative glycosyltransferase genes (*gtfB* and *vimF*) (Muthiah et al., 2013; Yamaguchi et al., 2010). Interestingly, the *pgn_0361* gene identified in this study was also annotated as a putative glycosyltransferase 2–3 domain in a previous study (Klein et al., 2017). Similar to the *gtfB* and *vimF* mutant strains, the *pgn_0361* mutant strain also exhibited enhanced biofilm formation. Moreover, Shoji et al. (2018) designated the PGN_0361‐encoding gene as *gtfC*, which is involved in the biosynthesis of the O‐side chain of A‐LPS. The *gtfC* mutant exhibits A‐LPS deficiency, which might lead to the loss of colony pigmentation or hemagglutination.

In our study, the activities of Rgp and Kgp in the culture supernatant of the *pgn_0361* mutant strain were significantly higher than those in the culture supernatant of the wild‐type strain, whereas these activities in the cell pellets were significantly less than those of the wild‐type. Shoji et al. (2018) have also shown that the cell‐associated gingipain activities of *gtfC* (PGN_0361) mutant strain were reduced whereas most of its gingipain activities were observed in the supernatant. Similar observations have been reported for other gingipain regulatory genes, such as *porR* and *gppX* (Hasegawa et al., 2003; Shoji et al., 2002). These findings indicate that these mutant strains were able to produce Rgp and Kgp but could not retain them on the cell surfaces. It is known that both Rgp and Kgp belong to a group of C‐terminal domain (CTD) proteins, which are attached to the cell surfaces through the modification by anionic lipopolysaccharide (A‐LPS) (Gorasia et al., 2015). Since both *porR* and *gtfC* mutant strains were shown to be deficient in A‐LPS (Shoji et al., 2002, 2018), it is likely that these strains, including the *pgn_0361* mutant strain, failed to anchor the produced gingipains to the outer membrane of the cells due to the lack of A‐LPS; thus, the majority of gingipains was detected in the culture supernatant instead of the cellular fraction. However, our results did not concur with the report of Klein et al. (Klein et al., 2017), where the culture supernatant of the *pg_0264* (a *pgn*_0361 homolog) mutant strain, constructed in strain W83, did not exhibit any Rgp and Kgp activities. In ours and the study of Shoji et al. (2018), the mutant strains were constructed in strain ATCC33277. Therefore, the discrepancy might have resulted from strain variation. Previously, it has been shown that the distribution of Rgp and Kgp in various cell fractions of *P. gingivalis* was different in different strains (Potempa et al., 1995; Shoji et al., 2002).

In addition to characterizing the *pgn_0361* deletion strain, we also investigated the role of *pgn_0361* in the interaction between *P. gingivalis* and epithelial cells *in vitro*. The *pgn_0361* deletion mutant could not detach the epithelial cells and exhibited attenuated ability to inhibit wound closure. However, the culture medium of the mutant strain exhibited the strongest inhibition of cell migration. Arg‐specific and Lys‐specific gingipains are reported to mediate the inhibition of wound healing caused by *P. gingivalis* (Laheij et al., 2015). This may be due to the high concentration of gingipains detected in the medium of the *pgn_0361* deletion strain. Our results further demonstrated that the released gingipains in the culture medium of strain PgΔ0361 could actively inhibit the wound healing process during the entire test period (20 h).

The PgΔ0361 and wild‐type strains exhibited a significant difference in the inhibition of wound closure only at MOI of 100. At higher MOI, the levels of wound closure inhibition were similar in the PgΔ0361 and wild‐type strains. Laheij et al. (2015) reported that the *kgp* and *rgpArgpB* mutant strains at MOI of 1,000 exhibited a significantly lower inhibition of wound closure than the wild‐type strain. The PgΔ0361 cells may have residual gingipains, whose activity was too low to be detected using the fluorogenic probes. When the bacterial counts were high enough, these residual gingipains could be sufficient to inhibit the wound closure. In addition to gingipains, other factors, such as capsular polysaccharide (Laheij et al., 2015) in the PgΔ0361 cells, may also be involved in the inhibition of wound closure. Previously, the non‐encapsulated *P. gingivalis* strain was found to inhibit the wound closure more strongly than the encapsulated strain. The deletion of a glycosyltransferase may result in a loss of capsule, thereby inhibit wound closure even when the gingipains were absent (Davey & Duncan, 2006).

Oral diseases, such as periodontitis or mucositis, are infectious diseases involved host tissue destruction and regeneration, where periodontal pathogens played an important role in modulating tissue homeostasis (Silva et al., 2015; Smith et al., 2019). Cell detachment assay and wound healing assay have been used *in vitro* to investigate the influence of risk factors on tissue homeostasis and the intervention strategies (Laheij et al., 2015; Pastar et al., 2008). Hence, this study chose these two assays to investigate the role of *pgn_0361* in the interaction between *P. gingivalis* and the host. The host inflammatory response to microbes is also a critical host factor for the development of the infectious disease. The role of pigmentation‐related genes in this inflammatory process is certainly worth further investigation.

## 5 CONCLUSIONS

This study confirmed that the *pgn_0361* gene of *P. gingivalis* is involved in pigmentation and gingipain regulation. Additionally, this study demonstrated that the deletion strain exhibited reduced hemagglutinin activity and enhanced biofilm formation capacity. Moreover, the *pgn_0361* deletion strain also exhibited reduced virulence via its reduced ability to detach the adherent epithelial cells and inhibit wound closure. However, the culture medium of the mutant strain exhibited enhanced inhibition of wound closure, which may be due to high gingipain activity in the medium.

## CONFLICT OF INTEREST

None declared.

## AUTHOR CONTRIBUTIONS

**Hongyan Liu:** Data curation (equal); formal analysis (equal); funding acquisition (equal); investigation (equal); methodology (equal); software (equal); visualization (equal); writing – original draft (equal). **Lijia Huang:** Data curation (equal); formal analysis (equal); investigation (equal); methodology (equal); software (equal); validation (equal); visualization (equal); writing – original draft (equal); writing – review and editing (equal). **Yanling Cai:** Data curation (supporting); formal analysis (supporting); funding acquisition (equal); investigation (supporting); software (supporting); writing – review and editing (supporting). **Floris J. Bikker:** Formal analysis (supporting); methodology (equal); validation (equal); writing – review and editing (supporting). **Xi Wei:** Conceptualization (lead); funding acquisition (lead); project administration (lead); resources (lead); supervision (lead); writing – review and editing (equal). **Dong Mei Deng:** Conceptualization (lead); formal analysis (lead); funding acquisition (equal); investigation (lead); resources (lead); software (equal); visualization (equal); writing – review and editing (equal).

## ETHICS STATEMENT

None required.

## Data Availability

All data are provided in full in the results section of this paper.
